# Does suprascapular nerve block reduce shoulder pain following stroke: a double-blind randomised controlled trial with masked outcome assessment

**DOI:** 10.1186/1471-2377-10-83

**Published:** 2010-09-21

**Authors:** Zoe A Allen, E Michael Shanahan, Maria Crotty

**Affiliations:** 1 Division of Rehabilitation, Aged Care and Allied Health, Repatriation General Hospital, Daw Park, South Australia, 5043, Australia; 2 School of Medicine, Flinders Clinical Effectiveness, Flinders University, GPO Box 210, Adelaide, South Australia, 5001, Australia

## Abstract

**Background:**

Shoulder pain is a common complication of a stroke which can impede participation in rehabilitation programs and has been associated with poorer outcomes. The evidence base for current medical and therapeutic management options of hemiplegic shoulder pain is limited. This study will evaluate the use of suprascapular nerve block injection as part of an interdisciplinary approach to the treatment of shoulder pain following stroke. The technique has previously been proven safe and effective in the treatment of shoulder pain associated with rheumatoid arthritis and degenerative shoulder conditions but its usefulness in a stroke population is unclear.

**Methods/Design:**

A double blind randomised placebo controlled trial will assess the effect of a suprascapular nerve block compared with placebo in a population of 66 stroke patients. The trial will measure effect of injection on the primary outcome of pain, and secondary outcomes of function and quality of life. Measurements will take place at baseline, and 1, 4 and 12 weeks post intervention. Both groups will continue to receive routine physiotherapy and standard ward care.

**Discussion:**

The results of this study could reduce pain symptoms in persons with mechanical shoulder pain post stroke and provide improvement in upper limb function.

**Trial Registration:**

This trial is registered with the Australian New Zealand Clinical Trials Registry (ANZCTR) - ACTRN12609000621213.

## Background

In any year, there are approximately 48,000 stroke events amongst Australians. Shoulder pain is a distressing complication of hemiplegia [1] and is reported as one of the 4 most common medical complications of stroke [2]. The prevalence of shoulder pain following stroke has reported to be as high at 70% [3]. A more recent prospective study of 327 consecutive stroke patients concluded that almost a third of this population developed moderate-severe shoulder pain after stroke onset [4]. This more moderate figure reflects the 2006 paper by the same investigators, which focused on patient's perspectives on pain [5]. Each of these studies highlight a correlation between pain and reduced functional ability, as well as a higher incidence of depression. Hemiplegic shoulder pain is associated with reduction in functional use of the arm, interference with rehabilitation and increased length of hospitalisation [6]. A further complication of hemiplegic shoulder pain is identified as a limitation to patient access to developing technological upper-extremity rehabilitation techniques [7].

Investigation into the cause of hemiplegic shoulder pain has revealed a multifactorial aetiology [8]. Note is made of the dependence on musculotendinous integrity to provide stability of the shoulder complex. The most common non-central, musculoskeletal aetiologies of hemiplegic shoulder pain include adhesive capsulitis, subluxation and rotator cuff patholgies, with up to one-third of patients have multiple contributing factors [8]. Biomechanical changes result from a combination of paralysis, fluctuation in muscle tone and prolonged shoulder immobility which lead to postural malalignment [1]. Dromerick et al [7] investigated the characteristics of hemiplegic shoulder pain, demonstrating that approximately 50% of the sample population experienced pain in the vertical stabilisers of the shoulder (biceps and supraspinatus). A 2006 evidence-based medicine review concluded that subluxation may be a cause of shoulder pain [9], though literature is inconsistent regarding this association. It should be noted that not all shoulder pain is associated with the complications of limb flaccidity, and may be attributable to spasticity or central-pain concepts.

There is lack of evidence to support the development of clear clinical guidelines, as identified in an overview of the challenges of managing shoulder pain after stroke [10]. This paper concludes that further efforts are required to examine intervention options. There have been positive research results for the use of Functional Electrical Stimulation [9], though a Cochrane Systematic Review [11] of this topic did not support electrical stimulation as an effective pain treatment. There is a lack of Level 1 evidence for surgical interventions, motor blocks and intra-articular corticosteroid injection.

Suprascapular nerve block is a safe and efficacious treatment of shoulder pain associated with rheumatoid arthritis and degenerative shoulder conditions [12]. The objective of this study is to evaluate the use of Suprascapular nerve block as part of an interdisciplinary approach to the treatment of shoulder pain following stroke. There is anecdotal report of successful use of suprascapular nerve block in treating intractable hemiplegic shoulder pain [1], though to date no clinical trials have been completed to form an evidence base.

## Methods and design

**Figure 1:**
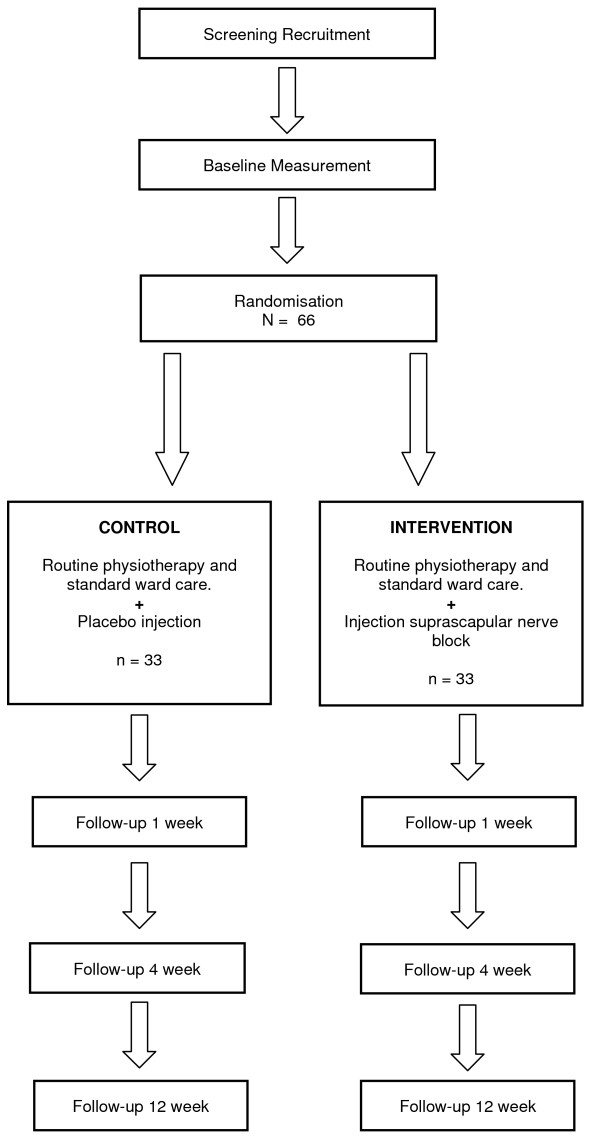
**Study Design - Randomised Controlled Trial**.

The study design is a double blind randomised placebo controlled trial will assess the effect of a suprascapular nerve block compared with placebo in a population of 66 stroke patients (Figure 1). The trial will measure effect of injection on the primary outcome of pain, and secondary outcomes of function and quality of life. Measurements will take place at baseline, and 1, 4 and 12 weeks post intervention. Both groups will continue to receive routine physiotherapy and standard ward care.

### Randomized controlled trial

#### Participants

Participants will be willing patients aged over 18 years with a diagnosis of acute stroke within the previous 12 months and onset of hemiplegic shoulder pain post stroke with a visual analogue scale score of > 30 mm (100 mm scale). Exclusion criteria will include the following:

▪ cognitive deficit that preclude patients from reliably using subjective outcome measures scales (Mini-Mental State Examination (MMSE) < 23)

▪ language deficits (inability to follow 2-stage command) or limited English language that preclude patients from reliably using subjective outcome measures scales

▪ allergy to proposed injection agents (depo-medrol 40 mg and 0.5% bupivocaine hydrocholoride)

#### Setting/locations

Participants invited to participate in the study will be recruited via the acute stroke and rehabilitation wards at multiple hospitals sites across Adelaide, South Australia including: Repatriation General Hospital, Flinders Medical Centre, The Queen Elizabeth Hospital, Hampstead Rehabilitation Centre (Royal Adelaide Hospital), and Griffith Rehabilitation Hospital. Ethics approval for the study has been granted by the Human Research Ethics Committees of Flinders Medical Centre (61/09), Royal Adelaide Hospital (09325), Repatriation General Hospital (09/09) and Queen Elizabeth Hospital (2009031).

#### Procedures

Participants will be assessed at baseline (following recruitment) and then at 1, 4, and 12 weeks following injection. In addition to demographics and classification of stroke, these four assessments will include the following measures:

(i) AbilityQ and ShoulderQ [13]

(ii) Modified Rankin Scale [14]

(iii) Croft Disability Questionnaire [15]

(iv) Euroquol [16]

(v) Visual Analogue Scale [16,17]

(vi) Application of 3 clinical tests shown to be predictive (98% probability) of hemiplegic shoulder pain [18]

Following consent and baseline measures, participants will be randomized to receive suprascapular nerve block or placebo injection. Allocation will be managed by a pharmacist external to the project.

#### Randomization

Participants will be assessed for eligibility, provided with information about the study, provide informed consent, be enrolled into the study and complete the baseline assessment prior to allocation into the control or intervention group. Participants will be assigned to the control or intervention group by a pharmacist external to the project by simple randomisation generated by a computer software program.

#### Intervention

##### Intervention Group

The intervention group will receive an a suprascapular nerve block injection to the back of the affected shoulder (using depo-medrol 40 mg and 0.5% bupivivocaine hydrochloride). The technique proposed for suprascapular nerve block [12] involves approaching the patient from posterior aspect of shoulder, which will ensure patient unable to visualise syringe contents. The doctor administering the injections will not be blinded for safety reasons. This approach has been used in a prior trial examining suprascapular nerve blocks [12] Intervention participants will continue to receive routine ward care of positioning of limb, careful manual handling and physiotherapy/occupational therapy suitable for the individual. The treating team will remain blinded to the randomisation.

##### Control group

The control group will receive an injection to the back of the shoulder of 5 ml normal saline infiltrated subcutaneously after the 2 ml subcutaneous 1% lidocaine infiltration. Control participants will continue to receive routine ward care of positioning of limb, careful manual handling and physiotherapy/occupational therapy suitable for the individual. This project does not involve the withholding of standard treatment to any participant. The treating team will remain blinded to the randomisation.

#### Outcomes

Outcomes will be assessed at 1 week, 4 weeks and 12 weeks by a physiotherapist blind to allocation. Proposed primary outcome measure involves use of a 100-point modified visual analogue scale (VAS) to assess pain [17]. This measure involves a 100 mm vertical line with periodic demarcations, anchored with the written extremes of subjective pain. Patients are asked to mark the severity of their current self-perceived pain on the scale, and this is then recorded in millimetre readings. Research suggests that a minimum change of 20 mm on the VAS is required to demonstrate clinically significant lessening of pain (initial reports >60 mm) [17]. Whilst a lesser minimum change is accepted for lower initial pain scores, we have chosen the stronger difference in the context of best evidence in a population who is predicted to report higher pain scores.

Secondary outcomes of disability and quality of life will be measured using the Modified Rankin Scale [14], Croft Disability Questionnaire [15], and the EuroQol Health Questionnaire [16]. The Croft Disability Questionnaire [15] includes 22 questions regarding disability associated specifically with shoulder pain. This measure is validated and chosen for this study as it more applicable in a more dependant sample population. Minimal level of detectable change (90% confidence) will be 3 points. Secondary outcome of spasticity will be measured using the Modified Ashworth Scale (MAS). MAS scores spasticity from 0-5.

Validity data will be collected for the AbilityQ and ShoulderQ measures [13]. These tools were developed by Lynn Turner-Stokes in 2006 to provide a sensitive measure of shoulder pain which is responsible to change in pain experience in a stroke population.

#### Sample size

Based on the data in the table 1[12], the standard deviation of the change scores are assumed to be in the range of 18-25. The attached table includes the estimated required sample size for a range of standard deviations and the three different clinically interesting changes above.

**Table 1:** Sample Size

Standard Deviation (change)	Treatment Change	N per group	N total
18	20	14	28
	28	8	16
	30	7	14

20	20	17	34
	28	9	18
	30	8	16

22	20	20	40
	28	11	22
	30	10	20

25	20	26	52
	28	14	28
	30	12	24

Hence using a conservative estimate, it is expected that a sample size of 26 participants per group (treatment and placebo) will achieve a statistically and clinically significant difference between the two groups (power 80%, alpha 0.05). To allow for deaths and withdrawals with a total attrition rate of 20%, a minimum total of 66 participants will be recruited, 33 per group. It is anticipated that recruitment of 66 participants (33 treatment, 33 placebo) size will take approximately 12 months and that each patient will be followed for 12 weeks.

### Statistical analysis

Data will be exported into SPSS software for subsequent analyses. A statistical analysis plan will be drafted at the start of the project and all analyses will be carried out after masking allocation.

The research questions will be assessed using an intention to treat approach. Independent samples t-tests, Mann-Whitney U tests and Chi-square test of association will be used as appropriate to compare groups at baseline. To determine differences between the groups at the primary end-point, ANCOVA or logistic regression will be used with models adjusted according to potential confounders.

## Discussion

The protocol has been carefully designed with the aim of achieving measurable, replicable and important results. The methodological strength of the study focuses around the use of placebo control, though the contributors acknowledge that this may pose a recruitment challenge. Considering that eligible patients have a pain score of > 3, it is anticipated that patients may decline participation on the grounds of not wanting to risk 50/50 chance of randomisation to placebo group. Taking this into account, greater time allowance has been given for recruiting. Careful provision of information prior to consent is vital in ensuring patient's are fully aware of implication of the randomisation. All patients will be informed of their randomisation group at the end of their trial participation and offered active suprascapular nerve block if desired.

Another uncertainty is in establishing methodology to catch probable timing of hemiplegic shoulder pain. Lindgren's 2007 population-based study on hemiplegic shoulder pain found that the majority of the incidence of pain occurred within the first 4 months post stroke [4]. Our inclusion criteria allow for patients to be up to twelve months post stroke, allowing for later incidences of pain occurrence. Difficulty may arise, however, in that ethics approval required injection in inpatient facility only. It is anticipated that many otherwise eligible participants may be unidentified by inpatient recruitment strategies.

Despite the realistic uncertainties outlined above, this study will provide useful information pertaining to an important topic. Shoulder pain is a common and debilitating symptom for a large number of people following a stroke and currently there is poor evidence regarding effective treatments. If the study shows that the suprascapular nerve block is efficacious in management of hemiplegic shoulder pain, it could potentially provide a new treatment option for stroke patients.

## Competing interests

The authors declare that they have no competing interests.

## Authors' contributions

ZA, MS and MC conceived the study, drafted the original protocol, and applied for funding. All authors read and approved the final manuscript.

## Pre-publication history

The pre-publication history for this paper can be accessed here:

http://www.biomedcentral.com/1471-2377/10/83/prepub
